# Between Voices and Silence: Indigenous Women and Sexual Offenses by Men Among the Arhuaco People

**DOI:** 10.1177/10778012231179216

**Published:** 2023-06-12

**Authors:** Edwin Rubio Medina, Luisa Castaneda Quintana

**Affiliations:** 1 Centre for Social Studies (CES), 37829 University of Coimbra, Coimbra, Portugal; 2 Faculty of Law, 5620 McGill University, Montreal, QC, Canada

**Keywords:** Arhuaco people, Indigenous peoples’ justice, Indigenous women, legal pluralism, sexual offenses

## Abstract

The field of legal anthropology has widely debated Indigenous Peoples’ justice practices. However, Indigenous Peoples’ legal perspective on sexual offenses remains understudied. In this respect, this article approaches the spiritual and political dimensions of the Arhuaco People's justice system to examine its procedures and sanctions. We want to understand how the Arhuaco People administer justice in cases where male community members are allegedly responsible for committing sexual crimes against women. During fieldwork in the Arhuaco territory, the authors employ methodologies drawn from the procedural paradigm-legal conscience studies as an interpretive framework to understand how Arhuaco women conceive legal phenomena.

## Introduction

The Colombian Constitution of 1991 granted a new legal status to Indigenous Peoples^1^ by recognizing Indigenous communities’ political, administrative, and financial autonomy (articles 286, 287, 328, 329) as well as the existence of Special Indigenous Jurisdictions, following the principles of cooperation and coordination with the ordinary (state) jurisdiction (articles 246 and 330). In addition, the legal recognition of “Special Indigenous Jurisdictions” entitles Indigenous Peoples to administer and run their justice systems as an expression of autonomy (Tomaselli, 2016).

To analyze Indigenous Peoples’ justice in Colombia, the authors present insights based on their ongoing experiences as Arhuaco people's legal advisors and 3 years of field research. The Arhuaco people, who name themselves the *Iku*, live in the traditional and sacred territory of the Sierra Nevada in northern Colombia, which is also home to the Kankuamo, Kogi, and Wiwa Peoples. The Arhuaco People are composed of around 50,000 members organized in 60 communities throughout Cesar, Magdalena, and La Guajira departments. They have preserved, to some extent, their language, *Ikun*, and they are known for their significant participation in political demonstrations demanding recognition and respect for their rights. As a result, the Arhuaco people maintain their administration of justice and are preoccupied with continuously enhancing their judicial system.

The Arhuaco people have considerable political strength and strong cultural ties. However, due to state jurisdiction standards, they have faced difficulties operating their justice system respectfully and strengthening their culture. For instance, community members expect offenders to be held accountable for their actions and receive the same punishment they would receive if they were dealt with by the “ordinary” (state) jurisdiction. Some of them, especially women, do not see their traditional practices as a practical path to obtaining justice. Instead, they demand that the state, represented by the Office of the Attorney General, assume jurisdiction of specific cases.

It means they enter into conflict with the Colombian state. Our objective is to examine how Arhuaco people resolve cases in which community members are allegedly responsible for committing sexual offenses. In this line, we highlight the challenges the Arhuaco community faces when male sexual offenses against women are committed, including the impunity of the perpetrator. An increasing lack of trust in male authorities overseeing those cases (who often disregard the victims’ versions of events) and the difficulty of having these cases processed in both jurisdictions, their own and the state's.

While research on Indigenous Peoples’ legal systems is broad, the Indigenous legal system's framing of and dealing with sexual offenses remains understudied (see Deer, 2019; Marchetti & Daly, 2016; Marchetti & Downie, 2014). The limited literature presents case studies of implementing Indigenous Peoples’ jurisdictions in their territories. In some cases, the lack of autonomy and legal, political, and social obstacles for Indigenous Peoples to exercise their justice (see de Sousa Santos, 2012; Goodale, 2007; Sierra, 2004; Yrigoyen, 2004). The rights of all parties to a fair trial and the ease to access the justice system (see Ruiz-Chiriboga, 2017). Scholars have also been interested in documenting and arguing about the ways Indigenous peoples achieve the recognition of their relatively autonomous legal jurisdictions. Also, communities structure these and how they eventually coordinate their work with the state jurisdiction (see Caicedo, 2012; Trujillo, 2012; Viaene & Fernández-Maldonado, 2018).

Empirical research on Indigenous Peoples’ experiences of autonomy in Latin America also abounds (Blaser et al., 2010; González et al., 2010; Gutiérrez, 2008; Postero, 2007; Rengifo et al., 2014; Tomaselli 2016.). In Colombia, publications on ethnic rights in the Constitutional Court are frequent (Bonilla, 2006; Borrero, 2014; Sánchez, 2000). However, violence against Indigenous women continues to be a marginal issue within this literature (there are notable exceptions: Bidaseca, 2011; Gonzalez, 2011; Piñacue, 2005; Santamaria et al., 2019; Ulloa, 2016). Nevertheless, academics have yet to address how complex it is for Indigenous Peoples to administer their justice in cases related to sexual offenses. The Arhuaco have had to navigate between ceding jurisdictional authority to the state and claiming and defending their autonomy and *Law of Origin*^2^ by the Colombian State law*.* The Arhuaco legal system is under constant pressure from some Arhuaco women—often supported by individual state agents—who reject impunity and demand justice.

This article aims to present new insights about Arhuaco women's and men's perceptions of “justice.” We discuss white-mestizo Colombian society's influences in delegitimizing the Arhuaco people's judicial practices. We show how some Arhuaco women perceive “justice” based on their political ideology, cultural traditions, and lineage. We draw particular attention to how Arhuaco women reflect on applying their notions of justice in cases involving male sexual offenses against women. This article is divided into five sections. The first describes the methods we used. The second section examines the spiritual and political entities, procedures, sanctions, and sentencing in the Arhuaco justice system. The third section discusses the ongoing political confrontation among Arhuaco about their justice system. The fourth presents Arhuaco women's perceptions of their community and their concepts of justice, specifically regarding sexual abuse cases. Finally, the last section illuminates how the Arhuaco women must constantly navigate inter-legality to claim justice for the sexual offenses they have endured.

## Method

This article adopts the “procedural paradigm approach” to uncover the legal consciousness of Indigenous women about sexual abuse. The procedural paradigm approach focuses on the context of the dispute process, identifying the various involved social actors’ interests and strategies, and conflicts are progressively resolved, managed, and confronted (Comaroff & Roberts, 1981). We do not conceive justice systems separate from social and cultural processes. Instead, we see these as interconnected, mutually interwoven. This entanglement of the practice of law with sociocultural realities is evident in how disputes eventually reach resolutions regarding litigants’ attitudes and cultural practices.

Nader and Todd (1978) argued that all societies offer individuals a range of alternatives to resolve grievances, such as avoiding confrontation, engaging in persuasion, or seeking forms of mediation, arbitration, and adjudication. In this essay, we mainly focus on the resolutions with legal consequences. Our approach is directly informed by “legal consciousness studies” (LCS) and follows an interpretive theory of law based on the scenarios that culturally determine the actors involved in legal conflicts. This method prioritizes the empirical individual_,_ especially those relegated to legal systems.

We characterize our approach within the micro-interactionist sociological theory (Collins, 1994) since it focuses on scientific observation and how individual consciousness becomes a social phenomenon. However, our adaptation of LCS has a critical component (Trubek & Esser, 1989). Its focus is on subaltern places and subjects, considering that everyday social practices lead to legality. In the case of Arhuaco women, when interpreting, they create their version of legality, which differs from the State model and the male vision of Indigenous law. It is moving away from a hierarchical and centralized version of legal dogmatism generally associated with a privileged status of race, class, sex, or geopolitical position.

Therefore, the legal consciousness approach analyzes legality and inter-legalities as a social phenomenon made up of what several community members say and do (Ewick & Silbey, 1998). In turn, such a theoretical construction is essential to comprehend the legal consciousness of community members, who adopt mutable and interchangeable strategies of acceptance, negotiation, and resistance.

During the present work, we resorted to both frameworks (the procedural paradigm by Nader and Todd (1978) and LCS by Ewick and Silbey (1998) to illustrate the legal consciousness of Arhuaco women, reflecting the intricate perceptions and practices advanced by women in both state and Indigenous jurisdictions. We consider Ewick and Silbey's (1998) classification when discussing legal conscience through this process. This classification includes three positions: before the law, with the law, and against the law. The first one, “Before the law,” implies recognition of the law's objectivity and neutrality. Here, people consider laws to be truly fair and equal for all.

In other words, people trust the legal system. The second classification includes “with the law,” where the law is perceived as a game of skills, where the most cunning triumph. Thus, the legal phenomenon is neither good nor bad, and people must survive it by implementing strategies, practices, and adaptations. The last one includes “against the law,” where the actors assume the law has internal and insurmountable structural flaws. In this way, people manifest an open contradiction. However, people's stances toward law can be very diverse. They can generate collective processes, collective emancipation on a smaller scale, individual, or nonconformity. We discuss these categories further in the section on *Inter-legality processes among Arhuaco women*.

Throughout our fieldwork, we held extensive conversations to understand the legal consciousness of Arhuaco Women concerning sexual abuse. We shared their day-to-day political and spiritual experiences, mainly by attending the community's word circles, ceremonies, and assemblies. In meetings convened at least twice a month for six months, we led discussions about the following substantial issues: (a) the Law of Origin. It consecrates the mandates of Mother Earth, establishes the rules of governance, and is the maximum guide and basis of the thought of the Arhuaco people; (b) the judicial situation of violators of the Law of Origin; and (c) cultural and political guidance for preserving their sacred territory. These assemblies and meetings took place in Nabusimake, Jewrwa, and Simonorwa (centers of great spiritual, political, and cultural importance among the Arhuaco people) between June and September 2017.

In 2020, we returned to the territory (Valledupar-Pueblo Bello) to conduct interviews with four Arhuaco women^3^ to deepen our understanding of the intricate political, social, and cultural relationships that sexual crimes affect within the community. While not in the field, we maintain a firm commitment to and close relationship with the Arhuaco people, supporting them professionally in their ongoing struggles. The results of our study are derived from these two trips to the Arhuaco territory, multiple virtual conversations with Indigenous leaders, women, and lawyers involved in the Arhuaco Commission of Justice (2018–2019), and an iterative collaboration on this text with Arhuaco authorities and national and international scholars.

We identify as trustworthy outsiders due to multiple factors, such as not speaking the Iku language, not staying constantly in the territory, and gender differences. However, we consider that this does not harm our position (Bucerius, 2013) Arhuacos are used to having external allies. In the particular case of women who have suffered sexual abuse, they assume that this article contributes to reflecting on and illustrating the phenomenon within-outside the community.

## Spiritual and Political Authorities in Arhuaco Society

Among the Arhuaco people, there are two types of authorities, the spiritual and the political. They denominate the first as *Mamos*, whose role is to preserve traditional knowledge and assure compliance with the Law of Origin. On this basis, the Mamos serve as traditional healers, spiritual guides, and community public affairs consultants. In the first capacity, they conduct healing processes by investigating the causes of the disorder according to the Law of Origin. The Mamos have a crucial mission to restore equilibrium when their people breach the Law of Origin—as this breach generates a spiritual disharmony transcending the material world (Suárez-Krabbe, 2016).^4^ In the Arhuacos’ cosmovision, the material world replicates the spiritual one (*Anugwe*). They associate committing prohibited acts with negative energies called *Anugwe Gunsinna* (Zalabata, 2008).

As spiritual guides, the Mamos provide spiritual appraisement over decision-making processes regarding political, social, and cultural affairs based on consultations with their gods and Mother Earth. They likewise determine the ritual of payments (*pagamentos*) that the Arhuacos should carry out to comply with the Law of Origin. These *pagamentos rituals* consist of compensating for the benefits we receive from Mother Nature and retributing to the Fathers of Nature's elements that allow the life of all societies, aiming at maintaining the natural balance.

Similarly, Mamos prepare future Mamos to ensure cultural survival by transmitting traditional knowledge between generations. Future Mamos are identified by checking newborns’ navels. Once the infant is recognized as Mamo, they wait until he ages seven or eight to perform an initiation ceremony. Mamos present several sacred objects to the child, expecting him to choose the correct objects to show his nature as a Mamo. Afterward, the child will learn that innermost knowledge lives within stones, forests, lagoons, and high mountains (Nevada, 1997).

In their capacity as community public affairs consultants, the Mamos’ role comprises taking part as representatives of the Arhuaco people in meetings with national authorities, the private sector, international agencies, and others. The Mamos serve mainly as counselors in Arhuaco society (Suárez-Krabbe, 2012), providing a bridge between the spiritual and physical worlds grounded in interpreting the Law of Origin*.* However, one major criticism of the institution of the Mamos is the impossibility of objecting to their decisions, which are supposedly based on the immutable Arhuaco ancestry and spirituality. In addition, the role of the Mamo is restricted to men only.

The political authorities among the Arhuaco people do not hold the same power within the community as the Mamos. Their role derives from the Colombian government's adoption of the modern conception of Indigenous reservations (*resguardos*)^5^ and the imposition of a legal entity and a political-administrative structure model in 1974. This model divides political authority into two administrative levels. The first is the board of directors, commonly known as ‘*las directivas*, elected by consensus between the Mamos and the remaining community members. The directors include a legal representative for the reservation (*cabildo Gobernador*), a general secretary, a prosecutor, and an accountant. The second administrative level is 60 councils replicating the same board structure across the Arhuaco territory. These councils represent villages or settlements throughout the departments of Cesar, Magdalena, and La Guajira. Remarkably, women are not part of this political institution.

The political and spiritual organization of the Arhuaco people, including how conflicts are solved within their society and the different dynamics of authority and kinship, provide essential context for our proposed case study of sexual offenses. However, women are not included in the Arhuaco justice resolution process in political and spiritual organizations. Therefore, the following section will present the adjudication and sentencing procedures of the Arhuaco justice system, all of which combine the Law of Origin with external institutions and features.

## The Adjudication Procedure and Regime of Sanctions for the Arhuaco People

The Arhuaco people have adapted their legal system to the state's requirements with two simultaneous procedures for judging reprehensible conduct, the Tina and *Tikrinu*. The *Tina* procedure is a physical inquiry conducted by political authorities. This process is similar to national legal systems of inquiry. The offended person or close relatives inform local authorities (*Zakuku*) of the situation. If this authority cannot solve it, the case is brought as a “second instance” to the board of directors members. They consult with the significant Mamos,^6^ seven older representatives, local leaders, and the Commission of Arhuaco Justice.^7^ The board of directors is responsible for collecting enough evidence and testimonies from those involved and ruling the case. To impose a sanction, besides considering the results of the Tikurigun inquiry, they also analyze aggravating circumstances, such as using weapons.^8^

Conversely, the Tikurigun procedure is a spiritual inquiry that emerged from the Law of Origin. A Mamo whose mandate is to identify what is causing disharmony in the territory carries out this investigation. Once the offender is identified, they conduct him or her to a *Kunkurwa*,^9^ where the inquiry is performed. Mamos believe offenses happen because both the victim's and perpetrator's families broke the Law of Origin, so they examine the families’ pasts to establish what led to those situations. This exercise helps the Mamos understand how to repair the fissure caused by breaking the Law of Origin, to restore harmony and, consequently, the well-being of the two families and the community. This family-based inquiry is an important feature that differs from the methods of the penal state system, which individualizes the aggressors without considering the social context underlying a case. Common minor offenses are interpreted only by the Mamo, while serious offenses prompt both physical and spiritual investigations (Gomez, 2015). Once both the spiritual inquiry (Tikrinu) and physical investigation (Tina) is completed, the directors decide based on both results.

However, predicaments and serious questions arise in the Arhuaco justice system. Sometimes, the Mamos’ decisions may be influenced by kinship and gender, favoring male voices and close family ties. For example, the women we interviewed alleged that the kindred bond of the Mamos with offenders often causes bias in judicial investigations. Like the investigation process, the Arhuaco regime of sanctions merges positive and customary law. If they are found to have violated the Law of Origin, particularly in severe cases such as homicide or sexual offenses, the responsible person falls into darkness.

This sentence is interpreted as a punishment of nine years of confinement in a house of reflection (*Casas de reflexión* or *GWAMɄNMɄKE*), a form of alternative, community-based detention. This sanction is based on an analogy of rebirth: 9 years stand for 9 months of pregnancy, the period an Arhuaco offender must endure to move forward from darkness and return to the light. However, the Arhuaco people face some difficulties in enforcing this sanction, as imprisonment may violate their customs, which will be explained further in the following section.

## How Do Arhuaco People Face Imprisonment?

Implementing the Special Indigenous Jurisdiction has shown that it has filled some gaps with mechanisms influenced by or borrowed from Western judicial systems. For instance, detention centers were constructed in several Indigenous territories in Colombia to solve the problem of enforcing imprisonment sanctions. Even though detention centers are presented as less invasive and assimilationist than prisons, they perform the same function. For example, some Indigenous Peoples, such as the Nasa people from the southwest of Colombia, call these places of confinement “houses of harmonization.” In the country's north, the Kogi, Kankuamo, Wiwa, and Arhuaco peoples refer to them as “houses of reflection.” In presenting detention centers as entities closer to Indigenous values than penitentiaries. State authorities pretend those centers result from an intercultural exercise that harmonizes externally imposed institutions with Indigenous customs, traditions, and values. However, our research shows that the prison system within the Indigenous territory is inconvenient and leads to other difficulties.

Although the penitentiary is a recent institution imposed by the state, the Arhuaco, among other Indigenous Peoples, have assimilated this foreign institution with their customs and traditions. These so-called “houses of reflection” are in Indigenous Peoples’ territories. Although the Ministry of Justice partially funds them, this economic support is usually short-term. Consequently, Arhuaco authorities often cannot afford the maintenance and management of the houses of reflection. Instead, they are merely staffed by part-time volunteers who must simultaneously tend their crops to provide for their families. As a result, prisoners frequently escape from houses of reflection. While in the house of reflection, there is no guarantee of daily meals and other essential services. Prisoners’ families or the local community are in charge of providing what they need. The buildings also do not have proper ventilation, lighting, or sleeping quarters. These conditions could be interpreted as violating international human rights standards for ordinary prisons, which are also broken in many prisons worldwide, including Colombia and the United States. These challenges are exacerbated by the long sentences required. As a paradoxical result of these shortcomings, these facilities do not qualify as prisons in the eyes of the government of Colombia, which would be necessary to access additional state funding.

An additional problem is the use of imprisonment as a political strategy. Some interviewees affirm that the use of houses of reflection seems to depend on the political interests of the local authorities of each community. Sentences do not always seem to be proportional to the crimes committed, and the decisions of the board of directors may not be justified by evidence, resulting in criticism that the judicial process is not transparent. For example, not a single prisoner has been found guilty of sexual crimes within the *resguardo*, most likely because this would lead to a social, cultural, and political loss of prestige for the community.

One Arhuaco woman stated in an interview that houses of reflection did not work as expected because they do not contribute *per se* to restoring natural harmony and Arhuaco justice. While they may detain Arhuaco persons in one place, they do not provide opportunities to reflect on the consequences of the action committed. She explained that when a negative mechanism, such as the houses of reflection, is incorporated within their territory, her people must counterbalance the lack of reflection with something positive to restore equilibrium. Therefore, houses of reflection must be accompanied by thoughtful-physical spaces or practices in which Arhuaco people traditionally reflect and meditate about their life worlds, such as the garden for men and the weaving of bags (*mochilas*) for women.

## Typology of Cases That Challenge Arhuaco Justice

Under Arhuaco cultural principles, there are two categories of infractions of the Law of Origin based on the severity of the behavior: those actions considered minor infractions and those that undermine the Law of Origin by breaking its cultural, social, and spiritual balance. In 2017, the Arhuaco Justice Commission conducted quantitative research to identify the infractions of the Arhuaco people from 2000 to 2017. Based on 94 registered cases, the study showed that the most recurring infractions were: crimes against physical integrity (40% or 42 cases), sexual offenses (22% or 22 cases), and unethical Arhuaco behavior (12% or 12 cases). Although sex crimes are not statistically the most recurrent cases, it is safe to assume that these are considerably under-reported, as this occurs in most societies. Homicide or personal injury cases are often more easily evidenced. In contrast, Indigenous women, in particular, may have difficulty making a public denunciation in a patriarchal society that tries to silence them.

In addition to the ubiquitous challenges of addressing sexual offenses, Arhuaco authorities must attempt to do so while facing discord from their community and state institutions. Sexual offense cases have provoked quarrels among several Arhuaco members who do not trust the impartiality of Arhuaco justice. They believe that their justice system leads to higher rates of impunity. They denounce *machismo* and kinship bias among leaders and the *Mamos* in charge of the inquiry, which, they argue, benefit the offenders.

Based on those grounds, some women leaders have requested that the state prosecutor's office investigate sexual offenses in the Arhuaco territory. In response to those demands, some Colombian judges and prosecutors have argued that the Indigenous justice system does not comply with the right to due process. Therefore, this claim threatens Arhuaco governance. The following section will introduce a case study on sexual offenses to demonstrate how these cases challenge the autonomy and day-to-day administration of justice for the Arhuaco people.

## Case Study of Sexual Offenses Collected in 2017

This section will show how Arhuaco justice operates when solving a sexual offense case through two emblematic cases registered during fieldwork and present significant strengths and weaknesses from the perspective of some Arhuaco members. Among the Arhuaco people, there are subtle classifications to differentiate those members of the community that strictly obey the Law of Origin and those who are more flexible with it. The ones who strictly obey are part of the vital core of the Arhuaco culture. In contrast, the second group is more open to Western customs and practices because of the proximity between cities and the Arhuaco territory. Furthermore, these cases may illustrate how consent and sexual maturity are interrelated in Arhuaca society.

In 2015, a 12-year-old Arhuaco girl and her family accused the girl's uncle, an Arhuaco far from the vital core of the Arhuaco culture, of having raped the girl several times. The uncle and the girl's aunt lived in the city of Valledupar when the girl was sent to live with them because her parents could not offer her educational opportunities in their village. When the family members realized the situation, they brought the case to the authorities in Jewrwa, their ancestral territory. Here, the authorities and local Mamo established the trial's rules to find the truth. The authorities in charge of the physical process (*Tikrinu*), who were all men and some of whom were relatives of the defendant, activated the procedure by receiving the girl's statement. Afterward, the authorities manifested their skepticism regarding her story, saying that she had been manipulated to discredit the good name of the accused man.

They decided that this case would follow the procedure for old family quarrels. Therefore, the Mamo subjected the girl to a process to decipher the truth of her testimony and sent her to a “house of reflection” where she could not eat for four days. When two of the girl's other uncles complained about this procedure, they were sent to different “houses of reflection.” At the end of the fourth day, the girl admitted that her account was false. Subsequently, the authorities issued an acquittal in favor of the defendant and later determined that the other uncles created chaos within the community. Thus, they were mandated to bring several objects to repair their *bunignmu* (to remedy their creation of chaos and disorder within an Arhuaco community). Initially, the girl's relatives cooperated. However, they soon concluded that they did not have enough guarantees for a fair trial, so they brought the case before the Prosecutor's Office.

From this case, some reflections arise. First, applying the principle of a fair trial within Arhuaco cosmovision is challenging, considering the parameters for investigating and solving a case, which includes kinship and family ties, culture, and political, traditional, and economic power relations. Second, sexual crimes are taboo; neither the authorities nor the Mamos uses the terms “sexual offense” or “rape.” Instead, they refer to negative energies and spirits that break the balance. Finally, while initially bringing the case to the Arhuaco justice system, the victim and her family concluded that spiritual reparation was not enough; they aspired to a process that ended with an exemplary physical punishment against the sexual aggressor.

The second emblematic case concerns a 14-year-old Arhuaco girl who attended a bicultural high school in Pueblo Bello. For several months during 2014, her teacher, recognized as a leader in the region, was having sexual encounters with her. According to her family, he had sex with her. When her family noticed the situation, they reported the case to the Arhuaco authorities of Pueblo Bello. In response, the Indigenous leaders opened the procedure and heeded the teacher's statement, in which he pled guilty.

The Arhuaco leaders decreed that the teacher be punished with eleven years of imprisonment in a house of reflection. They decided that the first two years of the sanction would be in absolute confinement to regain freedom while fully complying with the mandates of the Mamo in charge. However, Pueblo Bello is a mestizo town, and Arhuacos are not the majority. Therefore, they face serious challenges when carrying out their judicial procedures regarding autonomy, economic capacity, and logistics. They cannot administer houses of reflection within the municipality, which leads to noncompliance with the decision of the Indigenous authorities. The teacher still lives freely in the town, and he has not served any of his sentences. Given this situation, a female Arhuaco leader persuaded the girl's family to bring the case to the local municipal prosecutor, proposing that the Colombian state should investigate all sexual crimes. This Arhuaco woman is perhaps the most visible and respected defender of Arhuaco human rights, which she has championed for decades inside and outside the communities. She has been involved in national and international fora of Indigenous Peoples’ rights, where she has gained valuable experience in political advocacy and has been trained in the rights discourse. She believed that bringing the case to the local prosecutor would lead to more recognition of the rights of Arhuaco women, which from her perspective, were not being considered by the *directivas*.

Several local state prosecutors who advise Arhuaco women victims of sexual crimes to denounce Indigenous aggressors support this point of view. Although state prosecutors must navigate complicated legal jurisdiction matters to take on this type of case, they promise better results than those of the Special Indigenous Jurisdiction. However, no significant progress has been made in this specific case, and the offender remains free.

Regarding consent and sexual maturity in the Arhuaco society, in the first case, the 12-year-old girl is perceived as immature and incapable (by most societies and community members) of being aware of or able to begin sexual activities. In the opposite sense, in the second case, most of the elderly in the community assume that a 14-year-old girl is an adult and can have consensual sex.

This attitude would largely be reproached by Colombian society (Sánchez, 2010). These complex situations raise a new challenge for Arhuaco justice. Even though for many Arhuaco women, men and women are equal, the arrival in the Arhuaco society and embracing of concepts related to gender and rights forces them to deal with the development of women's rights recognized in international and national legislation. For example, the narrative of women's rights might imply dramatic changes in how the Arhuaco society conceives justice and equality across society, such as allowing women to participate in political and *Tina* and *Tikrinu* (judicial) processes. Indeed, Arhuaco people face challenges in administering justice regarding sexual offenses because some members of the communities, guided by kinship biases and machismo, oppose tough sanctions in these cases. Therefore, we will now consider the broader context of cultural practices, institutions, and customs to address these difficulties.

As described previously, the exercise of the Indigenous jurisdiction leads to many challenges, such as the distrust of the Arhuaco justice by some members whose sense of justice is influenced by the hegemonic society. In this context, especially in cases of sexual crimes, their acute perception of a lack of justice, truth, and reparation motivates them to resort to ordinary justice, ultimately delegitimizing their Indigenous peoples’ justice system.

Another challenge is bridging the distinct gaps between formal and Indigenous justice. Some of those gaps arise due to the state's lack of understanding and acceptance that Indigenous Peoples have their cosmovision, laws, customs, and traditions as distinct peoples. In this respect, to tackle those barriers and difficulties, especially regarding sexual offenses, a suggested solution might be built by the community based on their values, culture, traditions, worldview, customs, and uses, which make up an autonomous exercise of self-government and self-determination.

## The Ongoing Political and Cultural Confrontation Among Arhuaco People Affecting Their Justice System

Before considering the Arhuaco women's perspectives regarding Arhuaco justice, particularly in sexual abuse cases, it is crucial to understand the ongoing political confrontation among the Arhuaco people. In August 2020, a new board of directors was appointed. The new Arhuaco chief is a man who was previously sanctioned for abusing his underaged sister-in-law. This election has divided the Arhuaco people into two sides. One side is the new board of directors headed by the new chief and his allies. They assert that they were legitimately elected by a majority and thus recognize themselves as the political representatives of the Arhuaco people.

The other side includes the former board of directors and some traditional leaders, who argue that the board had agreed to suspend the election until the COVID-19 pandemic was over to guarantee the participation of all 60 communities. However, this agreement was broken. The former board of directors argues that traditionally the Arhuaco representatives must be elected not by majority vote but by consensus among Mamos, Zakukus, and local authorities. Therefore, the nomination of the new board of directors should ensure the participation of at least most of the communities in the General Assembly. Accordingly, this group decided to resort to state judges to resolve the internal governance disagreement. Finally, on February 16, 2022, the Constitutional Court decided to suspend the nomination of cabildos of the Arhuaco people and considered that the Mamos, exercising their autonomy, appoint the new cabildo of the Arhuaco people.

Back in 2020, the beginning of the political conflict among the Arhuaco people involved disputes over ancestral and traditional governance in contrast to forms of political decision-making closer to hegemonic, non-Indigenous practices. The involvement of the newly appointed chief in a case related to sexual abuse prompted some Arhuaco women to take a stance and strengthen their positions regarding justice and sexual crimes. In some cases, they took solid public stances, including two videos circulated in mainstream newspapers and social media platforms in Colombia.

The first video was made by Arhuaco women who rejected the nomination of the new chief as he presumably committed sexual abuse. In the video, which was in Spanish, they argue that Arhuaco women are the fundamental axis of the survival of their people and culture. They represent the territory, nature, and knowledge and are the life-bearers under the Law of Origin. Therefore, they consider that having a chief who violates the Law of Origin threatens their people's governance and women's integrity. The women argued that this nomination lacked legitimacy and harmed their Indigenous autonomy and jurisdiction. They predicted that, in consequence, sexual abuse and impunity would continue to grow in their society. In this analysis, we classify this perspective as a legal consciousness *against the law* (against the Law of Origin).

Several Arhuaco women supporting the new chief's appointment made a second video responding to this. Speaking in Iku with Spanish subtitles, they refer to themselves as the legitimate women leaders of the Arhuaco people and representatives of the Arhuaco Women Council. This entity was unknown before the video recording. Nevertheless, the authors need further information about it. These Arhuaco women disregarded the message of the women in the first video, framing them as nonlegitimate leaders. By publicly denouncing the internal matters of the Arhuaco people, the pro-chief women argued that the anti-chief women discredited themselves and their people, which ultimately breached the Law of Origin. The second group of women resorted to Indigenous tradition to legitimize their discourse, claiming to respect the *Zaku*^10^ and the dialogue in the *Ka’dukwu*.^11^ The Arhuaco people debate, talk, agree, and decide in these places. Consequently, their perspective can be classified as legal *before the law*.

These events coincided with our interviews with various Arhuaco women leaders recognized for defending the repositioning of women's roles within their society's political, social, and cultural spheres. Based on open dialogues and interviews with some Arhuaco women and those videos, we observe three different stances regarding Arhuaco justice and sexual abuse offenses within their society, which we discuss in the following section.

## Inter-Legality Processes Among Arhuaco Women

We incorporate the term “inter-legality” to understand Arhuaco women's perception regarding sexual offenses. This concept can be interpreted as the empirical dimension of legal pluralism (Sierra, 2004); this view privileges actors’ practices to make up multiple dimensions of the legal phenomenon. Moreover, these practices should be conditioned by the social, political, and cultural structure that leads to the generation of acceptance, negotiation, and resistance processes to the different justice systems.

We observed that depending on the women's political ideology, cultural origin, and lineage, they may have different perceptions of their participation within Arhuaco society and their justice system. We grouped such perceptions into three categories following Ewick and Silbey's (1998) classifications of legal consciousness. The first is *women before the law*. This category encompasses Arhuaco women primarily raised in a *Ka’dukwu* who speak Ikun and are immersed in the Arhuaco cosmovision and livelihoods. They also are part of a lineage that has traditionally exercised significant spiritual and political influence over the Arhuaco people. Such a status backs the preeminent position they have within their society.

Interestingly, the women interviewed assert that they have a crucial role given by the Law of Origin*.* Thus, women actively take part in their community. As for their participation in the imposed administrative-political organizations of the General Assembly or board of directors, these women claim they are involved and take an interest in it yet hold no political role. Notwithstanding, some Arhuaco women are increasingly taking part by having relevant positions within the administrative organization of the Arhuaco people. The group defends the harmonization of the man-woman relationship through collaborative practice (Tzul, 2020). This position is not centered on communitarian or decolonial feminism. Instead, it trusts that despite gender contradictions, the practice can transform the unfair situations suffered by women.

In addition, there are higher interests, such as cultural preservation and the defense of the territory. Thus, the premise endorses women's empowerment without a spiritual confrontation against Arhuaco men, as there is a clear definition of the roles of each gender in their Law of Origin. Such a stance has implications for the legal consciousness of Arhuaco women before the law—they abide by the directives of the Arhuacos’ spiritual and political authorities. Therefore, they are inclined to conduct their claims within the parameters of Arhuaco justice.

The second (*women with the law*) and third (*women against the law*) categories are associated with some Arhuaco women raised inside and outside the Arhuaco territory. They have been more in contact with non-Indigenous people and further exposed to Western discourses of feminism and conceptions of justice that differ from the Law of Origin. Some of these women do not speak Ikun (only Spanish) because they grew up outside their territory, did have a non-multicultural education—state education system, or were evangelized.

In the second category, *women with the law*, some Arhuaco women are mainly concerned with obtaining justice and expanding their participation regardless of jurisdiction. Instead, they occupy an intermediate ideological position between the before and against-the-law groups. Their political and legal position defends, in principle, the vital core of the culture of Arhuaca. They reject some procedures in the administration of justice, particularly in cases of sexual abuse. However, they tend to be nonconfrontational because they want to reach higher levels of power and participation within the political structure of the *resguardo*. They favor efficiency in the judicial system to reduce impunity. Finally, they maneuver between the narratives and judicial practices that best suit their interests.

Furthermore, in the third category, *women against the law*, some Arhuaco women claim participation within their society's political, judicial, and administrative organizations. They argue that Arhuaco women do not have a voice in the political sphere and do not take part but are mere spectators or relatives of the victims or offenders in the justice system. Instead, they attempt to make women visible by incorporating themselves in decision-making in the community and integrating into their discourse the precepts of liberal feminism reinforced with some values and principles of the Law of Origin.

In this section, different forms of legal consciousness adopted by some Arhuaco women were identified based on their reasoning about justice, particularly in cases of sexual abuse, which is influenced by their political, social, and cultural position within the Arhuaco society. The different legal consciousnesses among Arhuaco women show how they practice inter-legality by (a) endorsing their justice system and being faithful to their Law of Origin; (b) resorting to the state system as a more effective avenue for justice, but maintaining their Arhuaco cultural and spiritual values, and (c) openly denouncing their exclusion from internal participation and demanding more inclusive decision-making processes founded on more mainstream feminist arguments.

## The Urgent Aspiration for Justice Among Arhuaco Women

The analysis of the houses of reflection shows that although the practicalities of managing detentions of Arhuaco people in their communities are challenging and may signify limits on autonomy and Law of Origin, the houses of reflection function as inter-legal hybrids amalgamating the Law of Origin and State Law. Moreover, the fact that such legal hybrids have been developed poses the question of why such hybrids or innovations in the Law of Origin cannot be developed to address sexual violence against Indigenous Women.

The response to these questions may rely on the different perceptions of justice that the Arhuaco women have. The case study and the insights from Arhuaco women show that, depending on where they are positioned, they have a different perception of justice and a distinct role within it, which indicates the complexity of administering justice within Indigenous territory to confront sexual abuse.

Interestingly, among Arhuaco women, we have observed that, on the one hand, there is a position that shields and adheres to their law and justice systems founded on the Law of Origin and its interpretation by legitimate authorities. They conceive their justice as a complex and holistic response involving cultural recovery, spiritual-physical healing of the territory, and strengthening harmony between Arhuaco men and women. Accordingly, they uphold that the Arhuaco authorities should resolve all matters.

We have proposed an analysis of the legal consciousness of Indigenous women in the face of sexual crimes. This approach fluctuates and crosses both jurisdictions (Colombian and Arhuaco laws) and implies certain practices that we characterize as inter-legal. This article attempts to identify the relationship between the social, cultural, political, and gender conditions that produce this legal consciousness and the ways of seeking justice to fight impunity for these crimes (in both jurisdictions). On the other hand, the Arhuaco women, who are apt to either resort to the state system for justice or denounce their exclusion from internal participation, argued that the Arhuaco justice properly functions when resolving minor conflicts related to the land, family matters, and thefts. However, these women claim that the Arhuaco justice system does not have the means to resolve sexual abuse cases. Instead, they believe that the offender and authorities hide behind being Indigenous to handle the cases internally.

They conceive that sexual abuse and rape are not part of Arhuaco culture but a consequence of interaction with the non-Indigenous society and that these behaviors are unacceptable. Precisely, sexual crimes are among the most severe offenses.

They appeal to the state's jurisdiction to resolve cases of sexual abuse because of the impunity and lack of justice resulting from the Arhuaco customary procedures and the disregard for women's rights in Arhuaco society. In addition, some male Arhuaco authorities even state that women are guilty of inducing men to rape them. The women in these categories of legal consciousness stress that this patriarchal attitude and procedural bias will increase mistreatment, violence, and abuse if sexual abuse cases are not stopped.

These Arhuaco women encourage other fellow women to resort to the state jurisdiction to investigate sexual abuse as a last resort due to the lack of results within the Arhuacos justice system. They both agree that when a sexual abuse case is adequately resolved within the Arhuaco system, meaning there is justice for victims, it will help them to regain confidence and belief in their justice system. This account of the Arhuaco women's perception of and claim for justice allows us to observe some difficulties when exercising justice, which is more difficult nowadays due to the cultural and political internal conflict among the Arhuaco people. Therefore, this ongoing conflict makes it more challenging to have a collective dialogue and a communal approach to find a standard ground solution to address sexual violence.

However, it also represents an opportunity of considering that all three positions have something in common: the respect for the Law of Origin and the aspiration to obtain justice from their legitimate authorities. Indeed, it is impossible to avoid external influences that shape their discernment about justice. In line with this, some Arhuaco women argue that the state should respect the autonomy of its justice system. However, it is possible to envisage future inter-legal practices that would strengthen Arhuaco's autonomy and justice system.

## Conclusions

This article has focused on the Arhuaco people of Colombia and their use of customary law to address sexual offenses. We have discussed how the Arhuaco justice system has been reshaped because of the dominant society's direct legal-institutional imposition or influence. We have looked at how such syncretism has brought about clashes of jurisdictions when administering justice. Some members have developed a perception of justice at odds with their Law of Origin.

Further, we addressed how these conditions sow internal and external discord, as state institutions are incompatible with Arhuaco's cosmovision. For instance, the traditional figures of Mamos believe that accurate judges live in nature. Moreover, the Arhuaco people have struggled to preserve their identity and administer justice while attempting to respond to internal and external pressures.

We have also highlighted the predicaments and questions raised by the Arhuaco jurisdiction regarding biases of power, gender, and kinship that affect their judicial decisions. Additionally, Arhuaco authorities have faced pressure from their members and the local prosecutor's office to comply with the right to due process, especially in serious crimes such as rape and homicide.

As a result, although Indigenous authorities want to preserve their identity and traditions as distinct people, they have yielded to the demands of the state in several aspects. For instance, if they adopt external institutions into their customary practices, Indigenous Peoples expect the state to legitimize their self-governance. However, this should not allow for the invisibilizing of cases of sexual violence against Arhuaco women nor impunity for perpetrators of sexual violence. Therefore, Indigenous Peoples and the state must understand inter-legality and eliminate impunity in both jurisdictions.

The case studies reveal how the Arhuaco people administer their conventional system concerning sexual offenses. These cases show how the different approaches to justice have led to clashes of jurisdiction. Specifically, in terms of sexual abuse, we cataloged three legal consciousness perspectives on Arhuaco women. According to the first, *before the law,* Arhuaco women obeyed it and trusted their political and spiritual authorities. For the second, *with the law,* we distinguish some women promoting a conciliatory, nonconfrontational position, enhancing the notions of communal feminism.

In comparison, other women invoke hybrid feminism combining Western theories and values of women's empowerment with the Law of Origin principles*.* The third category, *against the law*, relates to women claiming participation within their society's political, judicial, and administrative organizations. Therefore, they demand justice, resorting to state jurisdiction because of the lack of trust in the Arhuaco system. These categorizations are fluid, and the practices implemented by women can combine or alternate between these three perceptions, but there could also be other additional perspectives.

It is evidence of the mutability and fluctuation of legal consciousness and raises the intricate forms of inter-legality within the Arhuacos. Such legal consciousness of Arhuaco women's perception of justice reflects some difficulties when exercising justice but also shows some opportunity considering the common aspiration of justice and endorsement of their Law of Origin*.*

## Notes

1. We capitalize the wording “Indigenous Peoples” as per new indications from the United Nations Permanent Forum on Indigenous Issues and UNESCO; to reflect that the term encompasses a bundle of rights. It is the ongoing dialogue at the United Nations level on how Indigenous Peoples are progressively being capitalized as part of the renewed impetus on guaranteeing the fundamental human rights for critical segments of the population that feel marginalized or overlooked by other segments of the population.

2. The Law of Origin is the natural and ancestral law according to which every natural element has an established role. It is the foundation of the Arhuaco people. The Law of Origin comprises all the world’s knowledge and grants all beings functions, rights, and co-responsibilities. It guides the management of the ancestral territory and the preservation of cultural knowledge. The transgression or breach of the Law of Origin weakens the natural functions of the universe, the territory, and customary practices.

3. All names of the Arhuaco women we interviewed are confidential, given the sensitivity of the ongoing political context of the Arhuaco people.

4. One of the Mamo’s roles is to restore the balance between positive (*Anugwe Duna*) and negative energies (Anugwe Gunsinna). As a result, the Mamos reestablish the physical and spiritual balance of both the aggressor and the victim; these circumstances are comparable to diseases arising from non-payment to the spiritual beings of the Sierra Nevada (Zalabata, 2008).

5. The *resguardos* is the official delimited and titled territory granted to Indigenous Peoples in Colombia. Usually, these are territories that Indigenous Peoples have used, occupied, and possessed for a long time, considered their sacred ancestral lands.

6. 64 significant Mamos are considered the wisest in the territory, responsible for maintaining the natural balance and preserving the Arhuacos’ Law of Origin.

7. The Commission of Arhuaco Justice comprises Indigenous leaders with experience in investigation and sometimes lawyers with expertise in Indigenous Peoples’ jurisdictions. Created in the 1980s, this commission has fostered the Arhuaco right through inquiry and analysis clash of jurisdiction and the adaptation of punitive sanctions.

8. The use of weapons within Arhuaco territory is considered to affect the territory’s harmony as a whole.

9. A *Knkurwa* is a ceremonial house where Mamos study and interpret the Law of Origin*,* and the new Mamos receive traditional and spiritual training. It is also where Mamos impart the full spiritual knowledge to women, men, and children. Every region within the Arhuaco territory has a *Knkurwa*, yet four *Knkurwas* hold the highest spiritual and traditional status.

10. *Zaku* literally means “Mother of Earth.” *Zaku* represents the spiritual and material Mother of all existing beings: trees, water, and wind. *Zaku* symbolizes the fertility and femininity that hold the equilibrium of the planet. *Zaku* is sacred territory.

11. A *Ka’dukw* is a sacred space where the Mamos make spiritual connections and consult with the spiritual parents of each element in nature and the universe. The Mamos make a spiritual and material signal with the beings of nature. Based on this spiritual consultation, the Mamos determine the guidelines for their people and communities. The *Ka’dukw* comprises stones where the Mamos, authorities, and other members of the Arhuaco people sit.
